# Molecular Characterization of *Corynebacterium pseudotuberculosis* Isolated over a 15-Year Period in Switzerland

**DOI:** 10.3390/vetsci8080151

**Published:** 2021-07-30

**Authors:** Jessica Schlicher, Sarah Schmitt, Marc J. A. Stevens, Roger Stephan, Giovanni Ghielmetti

**Affiliations:** 1 Institute for Food Safety and Hygiene, Section of Veterinary Bacteriology, Vetsuisse Faculty, University of Zurich, 8057 Zurich, Switzerland; jessica.schlicher@uzh.ch (J.S.); sarahnicole.schmitt@uzh.ch (S.S.); stephanr@fsafety.uzh.ch (R.S.); 2 Institute for Food Safety and Hygiene, Vetsuisse Faculty, University of Zurich, 8057 Zurich, Switzerland; marc.stevens@uzh.ch

**Keywords:** *Corynebacterium pseudotuberculosis*, biovar Ovis, MLSA, molecular characterization, caseous lymphadenitis, Switzerland, whole-genome sequencing

## Abstract

*Corynebacterium* *pseudotuberculosis* biovar Ovis is the etiological agent of the contagious and chronic disease caseous lymphadenitis (CLA) in sheep and goats. The economic impact of CLA in Switzerland remains largely unknown, and the transmission modalities, as well as the genetic diversity of circulating strains, are poorly understood. This work presents further characterization data for 215 *C. pseudotuberculosis* isolates from sheep, goats and a dromedary originating from Switzerland and the Principality of Liechtenstein, collected over a 15-year period. The isolates were classified into the two biovars Ovis and Equi, analyzed for the presence of the diphtheria-like toxin gene and characterized using MLSA. All sheep and goat isolates were classified as *C. pseudotuberculosis* biovar Ovis. The isolate from a dromedary was classified as biovar Equi. No isolates harboring the diphtheria-like toxin gene were detected. Phylogenetic analysis of the concatenated sequences of four genes revealed the existence of 24 clusters. There was no correlation between MLSA sequence types, year of isolation and the geographical origin of the isolates. These findings confirm the presence of several MLSA sequence types in the study area and over a 15-year period. Moreover, no sheep- and goat-specific MLSA sequence types were found.

## 1. Introduction

*Corynebacterium* (*C.*) *pseudotuberculosis* is a Gram-positive, facultative anaerobic, intracellular pathogen with worldwide distribution [1,2]. This bacterium primarily causes disease in horses and small ruminants such as sheep and goats with marked geographical differences on the host’s prevalence [3,4]. Several other species including cattle, camelids, llamas, buffalo, wild ruminants and rarely humans can also be infected [5,6,7,8,9,10,11]. In small ruminants, an infection with *C. pseudotuberculosis* leads to chronic pyogranulomatous lesions known as caseous lymphadenitis (CLA), characterized by abscess formation in superficial and visceral lymph nodes. The infection can generalize into different internal organs such as the lungs or the liver resulting in the visceral form of the disease [12,13,14]. Transmission primarily occurs after infection of superficial skin wounds with pus from abscesses that drain spontaneously [3,15]. In addition, the pathogen can also be transmitted through ingestion or direct contact with aerosols [14,16]. In cattle and horses, transmission by houseflies has also been described [17,18,19].

*C. pseudotuberculosis* can be distinguished into two biovars based on its ability to reduce nitrate. Nitrate-negative strains are classified as biovar Ovis and nitrate-positive ones as biovar Equi [20,21,22]. In sheep and goats, CLA is predominantly caused by biovar Ovis, whereas the nitrate-positive biovar is the causative agent of abscesses and ulcerative lymphangitis in horses and oedematous skin disease (OSD) in buffaloes [2,4,7,23]. Nevertheless, the biovars do not display species specificity for a certain host [24].

CLA is a major problem in countries with intensive small ruminant farming, such as Australia and New Zealand, causing considerable economic losses in ovine and caprine herds through reduction of wool, meat and milk production [2,3,25]. Previous research suggests an average prevalence of 26% in Australia [25], 34% in Portugal [26], 43.7% in Brazil [27] and 21% in Canada [28]. Vaccination programs have been described to lower the occurrence of CLA [25]. In several countries including Switzerland, no vaccines are licensed. The actual prevalence of CLA is presumably underestimated in many countries because it is not a notifiable disease and the infection often proceeds subclinically without any sign of abscesses [12,27].

*C. pseudotuberculosis* is considered an occupational zoonosis; fortunately, infections in humans are rare [29,30,31]. The majority of human lymphadenitis cases have been reported in Australia, affecting workers who have had regular contact with infected sheep [6].

Two major virulence factors, the phospholipase D and the mycolic acids of the cell wall surface, play a decisive role in the pathogenesis of the disease and enhance the organism’s ability to survive phagocytosis and multiply within macrophages [3,32,33]. The cell surface lipids may also contribute to the organism’s high resistance to environmental conditions. For instance, it has been shown that *C. pseudotuberculosis* can survive four months in shearing stalls and up to eight months in soil [2]. Additionally, *C. pseudotuberculosis* can be converted to diphtheria toxin producers through a beta-corynebacteriophage encoding the diphtheria toxin gene [34,35]. The diphtheria toxin is the major virulence factor of *C. diphtheriae*, which is the causative agent of the fatal human disease called diphtheria [36].

It has been shown that various molecular typing techniques including restriction fragment length polymorphisms [37], pulsed-field gel electrophoresis [38] and ribotyping [39] are useful for classifying *C. pseudotuberculosis* into two biovars but not for further characterization, especially among biovar Ovis isolates which display remarkable homogeneity. Guimarães et al. (2011) and a cross-country study conducted by Dorneles et al. (2012) used ERIC-PCR for molecular typing of *C. pseudotuberculosis* biovar Ovis strains and reported a good discriminatory power and typeability [40,41]. However, ERIC-PCR has some significant drawbacks such as limited repeatability, poor standardization and therefore poor comparability of typing patterns between different laboratories [42,43,44]. Molecular typing methods, based on the amplification of housekeeping genes, such as multilocus sequence typing (MLST) and multilocus sequence analysis (MLSA), provide good reproducibility, and the resulting data can be shared in public databases, making them a suitable tool for epidemiological studies [45,46,47]. In MLST, allele sequences at each locus are assigned numbers. Strains that have the same alleles at all loci are considered to belong to the same sequence type. MLSA is a more flexible alternative for comparing bacteria; here, the actual DNA sequences are used in the downstream analyses. To date, limited data are available on the performance and discriminatory power of MLST and MLSA in *C. pseudotuberculosis* [48], although MLST has been proven to be useful for the characterization of *C. ulcerans* [49] and *C. diphtheriae* [50], which are both closely related to *C. pseudotuberculosis*. Since a reliable and reproducible molecular typing method with good discriminatory power is needed for *C. pseudotuberculosis*, the performance of a sequence-based molecular typing method should be evaluated.

The aims of the present study were to assess (i) the phenotypic and genetic diversity of *C. pseudotuberculosis* isolates from sheep and goats in Switzerland and the Principality of Liechtenstein over a 15-year period, (ii) the epidemiologic relationships among isolates using an MLSA approach and (iii) to evaluate the presence of a diphtheria-like coding gene.

## 2. Materials and Methods

### 2.1. Bacterial Isolates

In total, 215 *C. pseudotuberculosis* isolates were collected over a 15-year period (2006–2020). Thereof, 152 (70.7%) originated from sheep, 60 (27.9%) from goats, one from a dromedary (0.5%), and two isolates (0.9%) were of unknown origin. The dromedary with visceral pseudotuberculosis belonged to a camel farm in Switzerland and was imported two years prior to the clinical signs from a traveling circus in Germany [51]. The majority of the isolates (46.5%; *n* = 100) originated from healthy slaughtered sheep and goats; of these, 67 showed lesions in the lung. Ten isolates (4.7%) originated from animals with lesions in the lung which were brought to the Section of Pathology, University of Zurich. One-third (*n* = 67) of the samples originated from animals that showed abscesses in superficial lymph nodes or the skin. For the remaining 37 (17.2%) isolates, no information about the characteristics of the lesions was available. The isolates originated from animals of 21 out of 26 cantons in Switzerland. Cantons most strongly represented were Grisons (*n* = 45; 20.9%), Zurich (*n* = 38; 17.6%), St. Gallen (*n* = 21; 9.8%) and Bern (*n* = 17; 7.9%). Four isolates originated from the Principality of Liechtenstein (1.9%). For 25 isolates, no information about their geographical origin was available. After identification with API Coryne (bioMérieux, Marcy-l’Etoile, France), all isolates were stored on cryopreservation beads at −80 °C. Reference strains of *C. pseudotuberculosis* biovar Ovis (DSM 20689) and *C. pseudotuberculosis* biovar Equi (DSM 7177) were included.

### 2.2. DNA Extraction

For DNA extraction, the isolates were cultured on Columbia Blood Agar with sheep blood (Thermo Fisher Diagnostics AG, Pratteln, Switzerland) and incubated at 37 °C for 48 h with 5% CO_2_. A commercial DNA extraction kit (InstaGene Matrix, Bio-Rad Laboratories, Richmond, VA, USA) was used according to the manufacturer’s instructions. The supernatant was collected, and DNA concentration was determined spectrophotometrically with NanoDrop 2000c (Thermo Fisher Scientific, Waltham, MA, USA). DNA samples diluted to a concentration of 10 ng/μL were stored at −20 °C until use.

### 2.3. Nitrate Reductase Gene

All isolates were screened for the presence of the nitrate reductase gene as previously reported by Almeida et al., using the primers narG_F and narG_R [20]. The DSM 7177 reference strain biovar Equi was used as positive control. The PCR mixture contained 5 μL of QIAGEN HotStarTaq mix, 1.5 μL H_2_O, 1 μM of each primer and 2.5 μL of the genomic DNA. Amplification was performed in a final volume of 10 μL using a thermal cycler (Applied Biosystems SimpliAmp^TM^ Thermal Cycler, Thermo Fisher Scientific) as follows: first denaturation step at 95 °C for 15 min, followed by 30 cycles of denaturation at 95 °C for 30 s, annealing at 58 °C for 30 s, extension at 72 °C for 90 s and a final extension of 10 min at 72 °C. The amplified products were analyzed with a capillary electrophoresis system (QIAxcel Advanced, QIAGEN, Hombrechtikon, Switzerland). In addition, the nitrate reduction reaction generated by the API Coryne (bioMérieux, Marcy-l’Etoile, France) test was retained as biochemical control for each isolate.

### 2.4. Diphtheria-Like Toxin Gene

All isolates were screened for the presence of diphtheria-like toxin genes by a conventional PCR using the primers PAC_F and PAC_R described by Mancini et al. [52]. A toxin-gene bearing *C. ulcerans* isolate was used as positive control. The PCR mixture contained 5 μL of QIAGEN HotStarTaq mix, 1.5 μL H_2_O, 1 μM of each primer and 2.5 μL of DNA template. Amplification was performed using the following protocol: 95 °C for 15 min, followed by 40 cycles of denaturation at 95 °C for 30 s, annealing at 54 °C for 50 s, extension at 72 °C for 30 s and a final extension of 10 min at 72 °C.

### 2.5. Multilocus Sequencing

**Figure 1:**
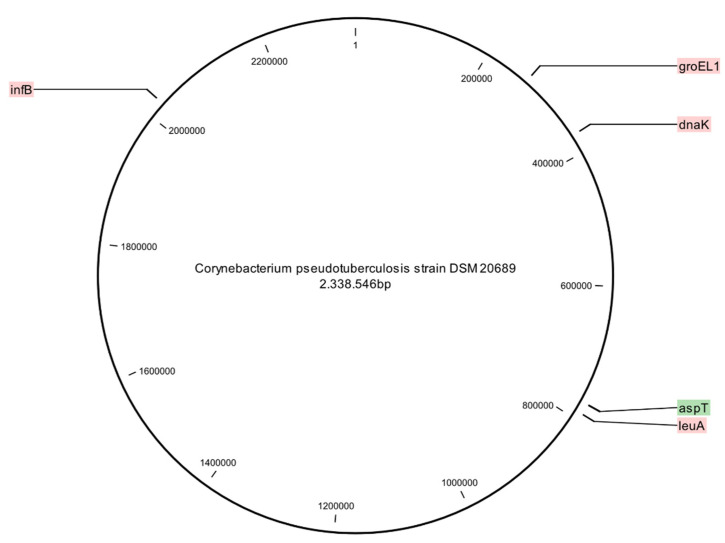
The location of the five genes used for the MLSA at the complete genome map of the reference strain of *C. pseudotuberculosis* biovar Ovis (DSM 20689).

A total of five genes including the heat-shock protein (*groEL1*), the translation initiation factor IF2 (*infB*), the 2-isopropylmalate synthase (*leuA*), the chaperone Hsp70 (*dnaK*) and an aspartate aminotransferase (*aspT*) were chosen for MLSA (Table 1) [48]. The location of the five genes in the genome of the DSM 20,689 reference strain *C. pseudotuberculosis* biovar Ovis (Acc. No.: NZ_RBXH00000000) is given in Figure 1. The following PCR cycle conditions were used: initial denaturation at 95 °C for 15 min, followed by 30 cycles (40 cycles for *aspT*) of denaturation at 95 °C for 30 s, annealing at 60 °C for 60 s, extension at 72 °C for 60 s (2 min for *aspT*) and a final extension of 7 min at 72 °C. PCR amplicon purification and sequencing were carried out at Microsynth AG, Balgach, Switzerland. Each PCR sample was prepared for sequencing using the gene-specific forward primer.

### 2.6. In Silico Sequence Analysis

The whole-genome sequences of nine *C. pseudotuberculosis* biovar Ovis strains (Supplementary Materials, Table S1) were retrieved from the NCBI database and compared to each other using the Whole Genome Alignment plugin (CLC Genomics Workbench version 21.0.1, QIAGEN) in order to identify additional discriminatory loci for genetic differentiation and molecular epidemiological investigations. Based on these results, a fifth locus, *aspT*, a gene that encodes an aspartate aminotransferase class I/II-fold pyridoxal phosphate-dependent enzyme, was added to the MLSA scheme.

### 2.7. Multilocus Sequence Analysis (MLSA)

The CLC Genomics Workbench software (version 21.0.1, QIAGEN) was used for phylogenetic analyses. The *dnaK* gene was excluded from the MLSA since no heterogeneity was observed in a selected panel of 100 isolates. The obtained gene loci sequences of the remaining four genes were trimmed, and the individual alleles were concatenated in specific order (*groEL1*, *infB*, *leuA*, *aspT*) to generate a 2577 bp long sequence. A phylogenetic tree was constructed for each gene as well as the concatenated gene sequence by UPGMA with 1000 bootstrap values. The modified phylogenetic tree was created with EvolView v2 [53], and goeBURST analysis was performed using PHYLOViZ 2.0 [54].

### 2.8. Discriminatory Index

The Hunter–Gaston discriminatory index (HGDI) was calculated according to Hunter and Gaston (1988) to evaluate the discriminatory power of the present MLSA approach. It was calculated separately for the MLSA compromising the already published genes, *groEL1*, *infB* and *leuA*, and for an MLSA including the new gene (*aspT*) [55].

### 2.9. Whole-Genome Sequencing

In order to evaluate the discriminatory capacity of the adopted MLSA scheme, six strains from three larger STs (“798”, “4394”, “1197”) with small genetic differences (one to four single-locus variants) in the MLSA were chosen and further typed by whole-genome sequencing. The strains were grown on sheep blood agar at 37 °C overnight prior to genomic DNA isolation using the DNA blood and tissue kit (QIAGEN, Hombrechtikon, Switzerland). The DNA was prepared using a Nextera DNA Flex Sample Preparation Kit (Illumina, San Diego, CA, USA) which produces transposome-based libraries that were sequenced on an Illumina MiniSeq Sequencer (Illumina, San Diego, CA, USA). Up to 16 sequencing libraries were analyzed on one lane, and demultiplexing and trimming were performed using the Generate FASTQ option in the MiniSeq Local Run Manager version 2.4.1. The sequencing outputs were between 137,893,014 and 195,969,406 paired-end reads of 150 bp, resulting in a genome coverage of approximately 55 to 80 times. The Illumina reads passed a quality check performed with FastQC 0.11.7 (Babraham Bioinformatics, Cambridge, UK), except for the “per base sequence content” check. The latter error caused by transposase-based libraries used in this study was ignored. Reads were assembled using SPAdes 3.13.1 in Shovill 1.0.4 (https://github.com/tseemann/shovill; accessed on 19 May 2021) using default settings. Shovill is a software package that automatically filters and trims reads and calculates kmers. The assembly was filtered, retaining contigs >500 bp, slightly below two times the read size (600 bp). Filtering two times the read size is a common cut-off in assemblies. For annotation, the NCBI prokaryotic genome annotation pipeline was used [56]. General information on the genomes is provided in Table S2 (Supplementary Materials).

All genome sequences are available in the NCBI GenBank database (http://www.ncbi.nlm.nih.gov/genbank/; accessed on 21 May 2021) under accession numbers JAHDUX000000000, JAHDUY000000000, JAHDUZ000000000, JAHDVA000000000, JAHDVB000000000 and JAHDVC000000000. Reads were submitted to the sequence read archive under bioproject number PRJNA731845.

#### Virulence Factor Analysis

Virulence factors were determined by a bidirectional best-hit comparison of the predicted proteome and the virulence factor core database protein set (http://www.mgc.ac.cn/VFs/; accessed on 13 July 2021), downloaded in July 2021. The predicted proteome was compared to the virulence factor database VFDB (reference https://academic.oup.com/nar/article/47/D1/D687/5160975; accessed on 13 July 2021). Comparison was done using BLASTP with a minimal of 75% coverage and a value of minimal 1exp-5.

## 3. Results

### 3.1. Biovar Classification

**Figure 2:**
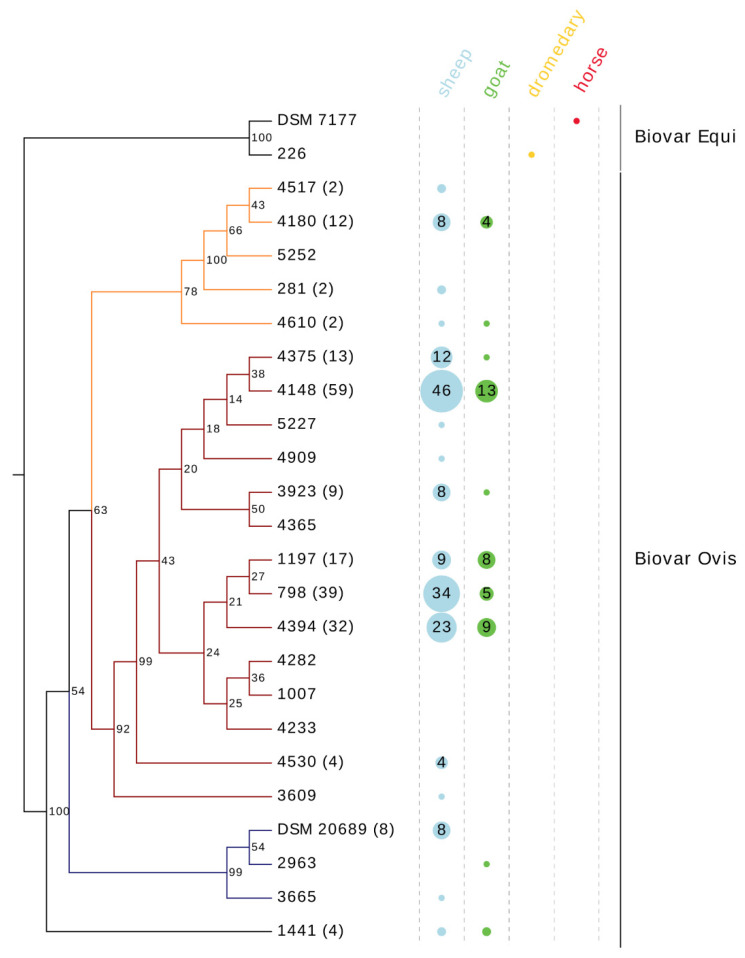
Modified UPGMA tree based on concatenated MLSA sequencing data for 213 *C. pseudotuberculosis* field isolates and two DSM reference strains. Two field isolates from unknown host origin belonging to the STs “4394” and “798” were excluded. The UPGMA tree was calculated using CLC Genomics Workbench with 1.000 bootstrap values. The tree was modified using EvolView v2. Branch colors indicate phylogenetic groups: orange—group 1; red—group 2, blue—group 3. The strain identifiers at the end of the branches indicate a representative isolate for each ST, in brackets the number of isolates belonging to the ST is given. ST “DSM 20689” corresponds to ST “4354” in the goeBURST diagrams. The columns show the host animals from which the isolates were isolated.

All isolates of ovine and caprine origin and the two isolates with unknown origin belonged to the biovar Ovis. The isolate from the dromedary was classified as biovar Equi (Figure 2). The biochemical nitrate reduction test and the PCR results for the nitrate reductase gene detection were consistent for all isolates.

### 3.2. Temporal Distribution

**Figure 3:**
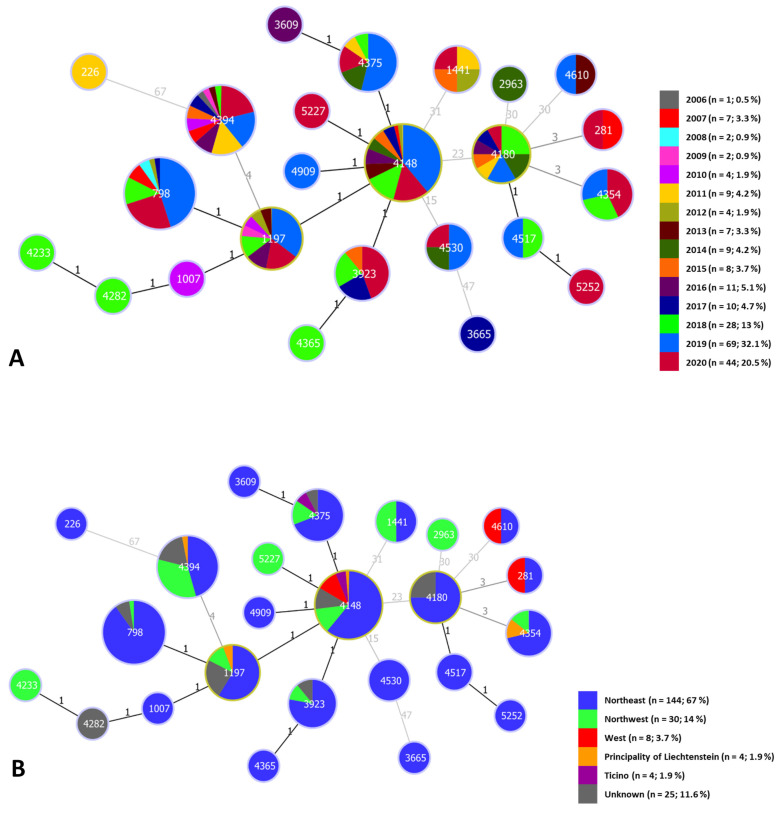
MLSA-based minimal spanning tree of 215 *C. pseudotuberculosis* field isolates. Two diagrams were calculated using the goeBURST full MLST algorithm in PHYLOViZ 2.0. Each circle represents one ST, and the size represents the number of isolates in each ST. Exact numbers of isolates per ST are given in Figure 2. Numbers within the nodes indicate a representative isolate in this cluster. Numbers on lines connecting STs indicate locus variants between nodes. Black lines indicate single-locus variants, and gray lines represent multilocus variants. (**A**) The isolates are color-coded according to the year of isolation, as given in the key on the right. The pie chart depicts the isolates from each year as fractions of the total number of isolates. (**B**) The isolates are color-coded according to the region of isolation, as given in the key on the right.

An average of six strains per year were obtained from 2006 to 2017, but when considering the entire study period (2006–2020), this number increases to an average of 15 strains per year (Figure 3A). The large increase of isolates in 2019 (*n* = 69; 32%) and 2020 (*n* = 44; 20.5%) is due to an additional project for which meat inspectors at slaughterhouses could send organs with lesions for further examination.

### 3.3. Allelic Variation

Three *groEL1*, 10 *infB*, 3 *leuA* and 5 *aspT* allele variants were obtained. All alleles of one gene had similar sequence lengths, apart from the *infB* gene alleles, for which sequence lengths ranging from 484 to 574 bp due to deletions and insertions of nucleotides were present. The *infB* gene displayed the greatest genetic variability among the analyzed genes. The minimal identity of the different alleles within *C. pseudotuberculosis* biovar Ovis varied within the data set presented here from 85% to 99%: *groEL1* 99%; *infB* 85%; *leuA* 99%; and *aspT* 99%. The total number of variable nucleotide sites among the biovar Ovis isolates was 199/2577 (7.7%); however, most of the variable nucleotide sites result from deletions and insertions in the *infB* gene.

### 3.4. Phylogenetic Analysis

The phylogenetic analysis of the 215 field isolates and the two DSM reference strains differentiated 25 sequence types (STs), 23 STs within the biovar Ovis (*n* = 215) and two STs within the biovar Equi (*n* = 2) (Figure 2). The two biovar Equi isolates clustered in a group more distant from the biovar Ovis isolates. Despite the relatively low level of genetic diversity, the biovar Ovis STs clustered in three closely related phylogenetic groups and one additional ST (“1441”), which was genetically more distant and comprised four isolates. Group 1 consists of 19 isolates and five STs. Group 2 comprises the majority of STs (*n* = 14) and shows very low internal bootstrap values, with the exception of two STs (“4530”, “3609”), which showed high internal bootstrap values. Group 3 consists of ten isolates and three STs. The most prevalent ST (“4148”) comprises 27% (59/215) of the isolates, followed by two STs, “798” and “4394”, containing 39 (18%) and 32 (15%) isolates, respectively. Together these three STs cover 60.5% (*n* = 130) of isolates included in this study. A total of ten isolates belonging to the biovar Ovis represent a unique sequence type each.

### 3.5. goeBURST Analysis

The association between the year of isolation and the geographic origin of the 215 *C. pseudotuberculosis* isolates in relation to their phylogenetic relatedness (Figure 3) was investigated. For the goeBURST analysis, the cantons of origin were grouped into three regions, except the Italian-speaking canton Ticino: northeast: Grisons (*n* = 45), Zurich (*n* = 38), St. Gallen (*n* = 21), Lucerne (*n* = 9), Thurgau (*n* = 8), Schwyz (*n* = 6), Schaffhausen (*n* = 5), Zug (*n =* 4), Appenzell Innerrhoden (*n =* 3), Obwalden (*n =* 2), Appenzell Ausserrhoden (*n =* 1), Glarus (*n =* 1) and Uri (*n =* 1); northwest: Bern (*n =* 17), Fribourg (*n =* 5), Aargau (*n =* 4) and Solothurn (*n =* 4); and west: Valais (*n =* 4), Vaud (*n =* 3) and Geneva (*n =* 1). This resulted in the following five geographical regions: northeast (*n* = 144), northwest (*n* = 30), west (*n* = 8), Ticino (*n* = 4) and the Principality of Liechtenstein (*n* = 4). The most prevalent ST “4148” persisted over the study period (2007–2020) and occurred in each geographical region of the present study. The oldest isolate, recovered from a goat in 2006, belongs to ST “4394” with 31 other isolates including 7 from 2020. The four isolates obtained from the Principality of Liechtenstein belong to different STs each. Temporal data or the geographic information did not show any clustering patterns.

### 3.6. Discriminatory Index

The Hunter–Gaston discriminatory index (HGDI) calculated for the MLSA using the genes *groEL1*, *infB* and *leuA* was 0.728; for the MLSA including the *aspT* gene, it was 0.853.

### 3.7. SNP Analysis of Six Sequenced Isolates

To confirm the MLSA results, six isolates belonging to three different major STs (“798”, “1197”, “4394”) were whole-genome-sequenced and compared using a whole-genome-based single-nucleotide polymorphism (SNP) analysis. The SNP analysis of the six isolates confirmed the high homology of the *C. pseudotuberculosis* biovar Ovis investigated in this study, with SNP numbers between the isolates ranging from 18 to 53 bp. The MLSA-generated STs were confirmed by the SNP analysis. Isolates “1197” and “4600”, grouped into the same ST, showed 18 SNPs difference, whereas the SNP differences between isolates “798” and “4480” and isolates “4394” and “3145” were of 23 SNPs and 24 SNPs, respectively (Supplementary Materials, Figure S1). The diversity between isolates of different STs ranged from 25 SNPs between isolates “798” and “3145” to 53 SNPs between isolates “4480” and “4600”.

### 3.8. Association between Sequence Type and Farms

With the information about the last owner of the animals, some isolates could be grouped according to their farm of origin. For a better epidemiological understanding, the owners of these flocks were contacted to obtain more information on whether the animals had had contact with other flocks. From 18 farms, two to six isolates were collected during the study period. In ten farms, all isolates collected belonged to the same ST. From one of these ten farms, six isolates sharing the same ST were obtained between 2012 and 2019. This flock had no contact with other animals in the last 21 years. Three additional herds in this group also had no contact with other animals; however, one owner reported purchasing sheep from different farms, and another one reported contact with one other flock when grazing in the summer months. In the other eight farms, two to three different STs were present. Interestingly, from two of these farms, four isolates from each farm were collected in 2019, and they belong to three different STs. Six owners in this group were contacted, four reported moderate to high contact with other herds, and two farms trade with sheep and/or goats.

### 3.9. Association between ST and Virulence

An association between the type of lesion and the STs was not found since the isolates from healthy slaughtered animals and from animals showing signs of disease were distributed in the larger STs. The six sequenced strains had also only a small difference in the virulence genes analyzed. All six strains had 95 virulence factors of which 94 were found in all strains. Strain 4600 lacked the periplasmic binding protein MntC (accession number VFG037082) but harbored an O-antigen export system permease protein (accession number VFG002227).

### 3.10. Diphtheria-Like Toxin Gene

Diphtheria-like toxin gene harboring *C. pseudotuberculosis* isolates could not be detected. Moreover, a BLAST search with the *C. diphtheria* toxin as query revealed that the diphtheria-like toxin was absent in the six sequenced genomes.

## 4. Discussion

In order to gain further information on *C. pseudotuberculosis* strains from animal infections in Switzerland and the Principality of Liechtenstein, a collection of temporally and geographically diverse isolates was further characterized. In the past decades, the genetic subtyping of *C. pseudotuberculosis* was a challenge. Many studies tried to differentiate this bacterium, using various molecular typing methods, without great success due to low genetic diversity of biovar Ovis [37,38,39]. With ERIC-PCR, some genetic differences within the biovar Ovis could be found [40,41]; however, this technique is hindered by limited repeatability and poor standardization [42,43,44]. Gene-by-gene approaches, such as MLST and MLSA, are one of the most important tools in modern molecular epidemiology; however, they have not been widely adopted for *C. pseudotuberculosis*, in contrast to *C. ulcerans* and *C. diphtheriae* [49,50]. Sellyei et al. (2017) established the first MLSA scheme for *C. pseudotuberculosis* and reported great genetic variability [48]. For the present MLSA, four genes (*groEL1*, *infB*, *leuA*, *dnaK*) of the before-mentioned study were retained and one new locus (*aspT*) was added to improve the discriminatory capacity of the analysis. This sequencing scheme was able to distinguish 24 STs among the 215 field isolates (Figure 2), in contrast to 15 STs, using only the three previously reported genes (*groEL1*, *infB*, *leuA*). Sellyei et al. (2017) analyzed thirteen *C. pseudotuberculosis* biovar Ovis field strains from Hungary and nine strains from the NCBI database; one DSM reference strain (DSM 7180) was also included in their analysis. They distinguished 23 STs among the biovar Ovis isolates [48]. However, it must be considered that the strains analyzed in the mentioned study originated from diverse geographical areas and various hosts, including buffalo, llama, antelope and human.

In the present study, the *infB* gene showed the greatest genetic variability, whereas a panel of 100 of our isolates did not show any genetic diversity in the *dnaK* gene. This finding is contrary to that of Sellyei et al. (2017) who reported the greatest genetic differentiation in the *dnaK* and the *infB* genes. Whether the *dnaK* gene shows higher genetic diversity in isolates from different countries and hosts needs further investigation.

The Hunter–Gaston discriminatory index (HGDI) is a key characteristic of typing systems, and it is used to measure the probability of two unrelated strains being differentiated. The calculated HGDI of 0.853 for the new MLSA scheme, compared to an HGDI of 0.728, using the already published genes from Sellyei et al. (2017), underlines the benefit through the new *aspT* gene for epidemiological studies of *C. pseudotuberculosis* biovar Ovis. Moreover, the index enables straightforward comparison between different typing methods. As before-mentioned, ERIC-PCR is another typing technique widely used to characterize *C. pseudotuberculosis*. The present MLSA and the ERIC-PCR 2 performed by Guimarães et al. (2011) show comparable discriminatory abilities (HGDI of 0.85 for MLSA and 0.87 for ERIC-PCR 2) [41]. However, MLSA has the advantage of producing reliable and reproducible results, which enables inter-laboratory comparison.

Lower genetic variability has been observed for *C. pseudotuberculosis* biovar Ovis compared to the biovar Equi [57,58]. MLSA revealed and confirmed a high level of clonal homogeneity of *C. pseudotuberculosis* isolates. Nevertheless, in the present study, 23 STs distributed over three phylogenetic groups and one outliner were identified among the biovar Ovis isolates. The small difference in the virulence genes analyzed in six sequenced strains and the distribution of the isolates from healthy and diseased animals in all larger ST groups suggests that the strain virulence is similar between STs and highlights the clonal-like behavior of *C. pseudotuberculosis* biovar Ovis.

Metadata analysis of the four isolates belonging to the outliner ST “1441” did not yield any epidemiological links between the isolates. The isolates were collected in four different cantons and years. Some of the smaller STs showed a similar pattern, especially ST “4610” regarding the geographic origin of the isolates and ST “281” regarding the year of isolation. ST “4610” consists of two isolates, one isolated in 2019 from a goat originating from the canton Vaud in the west of Switzerland and the other one isolated in 2013 from a sheep originating from the canton St. Gallen in the east of Switzerland. ST “281” consists of two isolates, one from 2007 and a second from 2020. To investigate whether these STs emerged independently in different regions or are the result of animal movements, deeper genomic analyses and more information about the animal’s history were required. However, the notification of sheep and goat movements in Switzerland only became mandatory in 2020. Thus, detailed movement data for the majority of the animals are missing.

In eight farms of the present study, up to three STs were found among isolates from animals of the same property. A possible explanation for this sequence-type diversity within a flock could be the way sheep and goats are usually kept in Switzerland. Some flocks spend the summer months grazing together with other flocks on Alpine and pre-Alpine pastures and return to their farms in the winter months. The result is a complex transmission chain between the flocks and could explain the occurrence of different sequence types within one farm. These results are in accordance with the observations of Guimarães et al. (2011) in Brazil, where up to nine genotypes per flock were found and were associated with intense animal movements [41]. This assumption is supported by the epidemiological data from the farms of this study where only one ST was detected. Four out of six herds had no contact with other animals, whereas the farms in which more than one ST was detected reported contact with other flocks or trading with animals. Additionally, isolates from sheep and goats did not show a species-specific clustering in the phylogenetic tree (Figure 2). These results are in agreement with earlier studies based on ERIC-PCR [40,41]. The present findings suggest that the chain of transmission is highly complex, and multiple factors may contribute to the large number of sequence types observed in some of the studied farms.

The analysis of the temporal data showed a constant presence of several clones of *C. pseudotuberculosis* biovar Ovis in Switzerland and the Principality of Liechtenstein, which can be seen as an example in ST “4394”, consisting of 32 isolates, including the oldest isolate recovered from a goat in 2006 and seven isolates from 2020 (Figure 3A). These findings combined with the SNP analysis of the six whole-genome-sequenced isolates performed in this study indicate a high degree of, respectively, genome conservation of *C. pseudotuberculosis* biovar Ovis and a permanent circulation of several STs in Switzerland. However, the introduction of new STs through import of infected animals may not be underestimated. For instance, isolate “3665” originates from a sheep, which was imported from Austria into Switzerland, and displays a unique ST in the present study. It differs genetically in 47 bp from the next closest isolates in ST “4530”. Thus, it shows the second-largest genetic diversity after the biovar Equi isolate, with 67 variable nucleotide sites differences from ST “4394”. However, the 47 variable nucleotide sites of isolate “3665” occur through a deletion in the *infB* gene, whereas the 67 variable nucleotide sites in the biovar Equi isolate correspond to a single-nucleotide polymorphism.

## 5. Conclusions

The good discriminatory ability of the adopted MLSA approach revealed several STs of *C. pseudotuberculosis* biovar Ovis in the Swiss small ruminant population. Over a 15-year period, little temporal and geographical variation of this pathogen was observed, highlighting a high level of clonal homogeneity. Animal movements between herds are assumed to cause disease transmission, and flocks affected by multiple *C. pseudotuberculosis* biovar Ovis strains were observed.

## Figures and Tables

**Table 1:** Oligonucleotide sequences of the primers used for amplification and sequence analysis ^a^.

Primer Name	Putative Gene Function	Primer Sequence (5′-3′)	Amplicon Size (bp)	Reference
*groEL1_F* *groEL1_R*	Heat-shock protein	ACCTTCACCGGATCATTG TTGGTGATCGTCGTAAAGC	655	Sellyei et al. 2017 [48]
*leuA_F* *leuA_R*	2-isopropylmalate synthase	AGCTCAGTGCGCGGTTGACC ATGGCGTCGCGGGTTCG	664	Sellyei et al. 2017 [48]
*infB_F* *infB_R*	Translation initiation factor IF2	ATTGCGGGACTTGGACG GCATTATGCTGCACAAGACG	642	Sellyei et al. 2017 [48]
*dnaK_F* *dnaK_R*	Chaperone Hsp70	TCCTTACCAGTGCCCTTATCC GAGTTCCAGCGCATCACC	580	Sellyei et al. 2017 [48]
*aspT_F* *aspT_R*	Aspartate aminotransferase	TCCCGCTGAGAACCTGGTTG GATCACTGCCTAGCCCACAT	1102	This study

^a^ F (forward) and R (reverse) primers.

## Data Availability

The original contributions presented in the study are included in the article/supplementary materials, and further inquiries can be directed to the corresponding author.

## Supplementary Materials

The following are available online at https://www.mdpi.com/article/10.3390/vetsci8080151/s1, Table S1: *Corynebacterium pseudotuberculosis* strains examined in the *in silico* sequence analysis; Table S2: General information on the genomes; Figure S1: Pairwise differences in the distance matrix of six whole-genome-sequenced isolates belonging to three different STs. References [59,60,61,62,63,64] are cited in the supplementary materials.
