# Supplementary material for: Molecular Characterization of Corynebacterium pseudotuberculosis Isolated over a 15-Year Period in Switzerland
Source: Vet Sci. 2021 Jul 30;8(8):151. doi: 10.3390/vetsci8080151 (PMC8402737; doi:10.3390/vetsci8080151)
Supplement: Supplementary file 1 [file vetsci-08-00151-s001.zip › vetsci-1274509-supplementary.pdf]

Supplementary material

Table S1. *Corynebacterium pseudotuberculosis* strains examined in the *in silico* sequence analysis.

Strain	BioSample	Accession Number	Reference
**1002**	SAMN02603609	CP001809	[59]
**C231**	SAMN02604117	CP001829	[59]
**T1**	SAMN04529096	CP015100	[60]
**42/02-A**	SAMN02603751	CP003062	[61]
**3/99-5**	SAMN02603750	CP003152	[61]
**29156**	SAMN03290960	CP010795	[62]
**PAT10**	SAMN02603741	CP002924	[63]
**Cp13**	SAMN04550073	CP014998	[64]
**276**	SAMN02603753	CP003407	[5]

Table S2. General information on the genomes.

Strain	N_{reads}[a]	Length (bp)	Contigs	N50	L50
**1197**	925,590	2321808	8	367418	3
**3145**	991,621	2320782	11	327282	3
**4394**	1,318,377	2320307	8	367274	3
**4480**	883,832	2320453	8	367420	3
**4600**	1,173,755	2320242	7	384780	3
**798**	974,433	2320469	8	367422	3

[a] refers to the number of reads in one set of the paired-end reads of 150 bp.

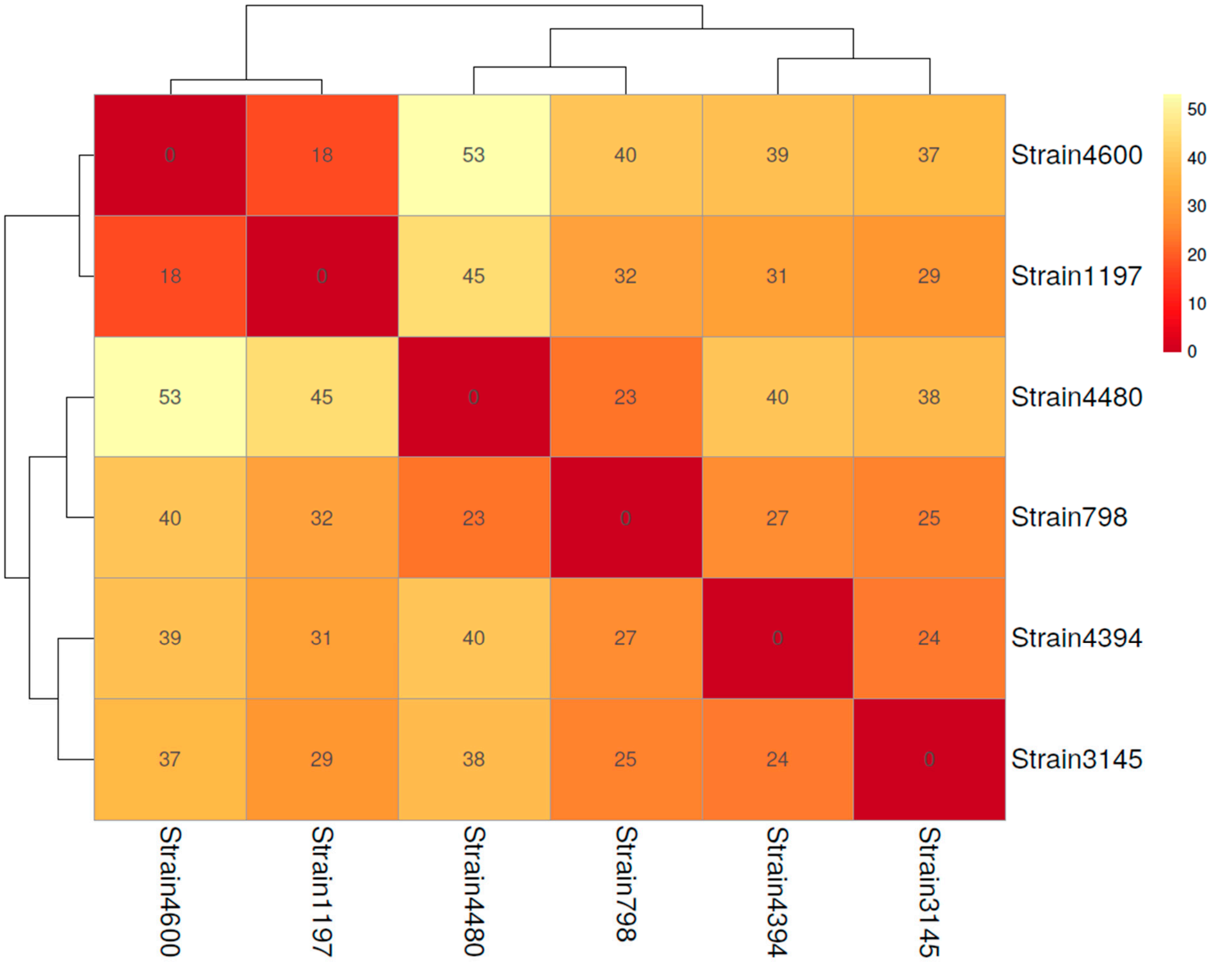

Figure S1. Pairwise differences in the distance matrix of six whole genome sequenced isolates belonging to three different STs.
